# Healthcare Service Use for Mood and Anxiety Disorders Following Acute Myocardial Infarction: A Cohort Study of the Role of Neighbourhood Socioenvironmental Characteristics in a Largely Rural Population

**DOI:** 10.3390/ijerph17144939

**Published:** 2020-07-09

**Authors:** Ismael Foroughi, Neeru Gupta, Dan Lawson Crouse

**Affiliations:** 1 Department of Sociology, University of New Brunswick, Fredericton, NB E3B 5A3, Canada; i.foroughi@unb.ca; 2 Health Effects Institute, Boston, MA 02110-1817, USA; dcrouse@healtheffects.org

**Keywords:** cardiac disorders, mental disorders, environment design, built environment, walkability, public health surveillance, rural population

## Abstract

Depression and other mood and anxiety disorders are recognized as common complications following cardiac events. Some studies report poorer cardiac outcomes among patients in socioeconomically marginalized neighbourhoods. This study aimed to describe associations between socioeconomic and built environment characteristics of neighbourhood environments and mental health service contacts following an acute myocardial infarction (AMI or heart attack) among adults in the province of New Brunswick, Canada. This province is characterized largely by residents in small towns and rural areas. A cohort of all adults aged 45 and over surviving AMI and without a recent record of mental disorders was identified by linking provincial medical-administrative datasets. Residential histories were tracked over time to assign neighbourhood measures of marginalization, local climate zones, and physical activity friendliness (i.e., walkability). Cox models were used to estimate hazard ratios (HRs) and 95% confidence intervals (CIs) for the risk of healthcare use for mood and anxiety disorders over the period 2003/04–2015/16 by neighbourhood characteristics. The baseline cohort included 13,330 post-AMI patients, among whom 32.5% were found to have used healthcare services for a diagnosed mood or anxiety disorder at least once during the period of observation. Among men, an increased risk of mental health service use was found among those living in areas characterized by high ethnic concentration (HR: 1.14 (95%CI: 1.03–1.25)). Among women, the risk was significantly higher among those in materially deprived neighbourhoods (HR: 1.16 (95%CI: 1.01–1.33)). We found no convincing evidence of associations between this outcome and the other neighbourhood characteristics considered here. These results suggest that selected features of neighbourhood environments may increase the burden on the healthcare system for mental health comorbidities among adults with cardiovascular disease. Further research is needed to understand the differing needs of socioeconomically marginalized populations to improve mental health outcomes following an acute cardiac event, specifically in the context of smaller and rural communities and of universal healthcare coverage.

## 1. Introduction

Cardiovascular diseases, including acute myocardial infarction (AMI, also known as a heart attack), are the leading cause of death worldwide; ensuring appropriate counselling and treatment for those having survived an AMI can help improve overall health and reduce the risk of another attack. In Canada, an estimated 578,000 adults have a history of a heart attack [1]. While medical advancements have led to improved prevention and management of AMI and other heart diseases, socioeconomic and other factors continue to drive disparities in the impacts of these conditions. There is growing evidence of poorer cardiac therapeutic outcomes among persons residing in lower-income neighbourhoods compared to those residing in more affluent neighbourhoods, even in the Canadian context of universal healthcare coverage [2,3]. Understanding the barriers and facilitators to accessing preventive and rehabilitative healthcare services can help reduce the risks of adverse outcomes for cardiac conditions and common comorbidities. In particular, depression and other mood and anxiety disorders are increasingly recognized as a complication of AMI [4,5,6], which can lead to increased risk of mortality [7,8,9]. Some research suggests that women may demonstrate a higher risk of anxiety or depression following an AMI compared to men [10]. Interventions to address systemic barriers to cardiac rehabilitation, such as lower health literacy, poorer social support, gendered differences in healthcare seeking behaviours, and factors inhibiting uptake of physical activity in everyday life, could have multiplicative benefits for reducing impacts of depression among patients who have suffered a coronary event [2,11,12].

Most of the available literature on neighbourhood-level socioenvironmental characteristics as predictors of health, including sociodemographic profiles of neighbourhood populations and characteristics of built environments, is based in larger urban areas [2,12,13,14] or in jurisdictions with a large supply of cardiac services [3]. Little is known about the role of neighbourhood environments in the context of uniquely small urban and rural areas, including the Canadian Maritimes. This region of Eastern Canada, largely characterized by numerous rural and ageing communities, consists of three provinces: New Brunswick, Nova Scotia, and Prince Edward Island. In New Brunswick, research has suggested that among adults living with a neurodegenerative disease, selected neighbourhood characteristics were related to increased risk of hospitalization for comorbid conditions including cardiometabolic diseases and mental health disorders [15]. No previous studies have examined socioenvironmental influences on mental health service contacts among those recovering from an acute coronary event. This study aimed to overcome this knowledge gap with the first Canadian investigation describing the associations between neighbourhood environments and the use of healthcare services for depression and other mood and anxiety disorders among patients surviving myocardial infarction. Specifically, a population-based observational cohort analysis was conducted using linked administrative and geospatial datasets to assess differentials in hospital admissions and physician visits for mood and anxiety disorders among post-AMI patients aged 45 and over in New Brunswick over the period 2003/04 to 2015/16 according to several physical and socioeconomic characteristics of local neighbourhoods.

## 2. Materials and Methods

### 2.1. Study Setting

From an international perspective, socioeconomic status in Canada is high, but New Brunswick presents poorer socioeconomic indicators than the national average (e.g., 17.1% prevalence of after-tax low income versus 14.2% nationally) [16]. With a total population of 747,100 according to 2016 Census data, New Brunswick is home to exclusively smaller cities and rural settlements. The province’s population is less ethnically diverse than the Canadian average (3.4% are visible minorities compared to 22.3% nationally) [16]. The crude AMI prevalence rate among adults was significantly higher in New Brunswick (3.08% (95% CI: 3.04–3.12)) in 2015/16 compared to the Canadian average (2.17% (95% CI: 2.16–2.17)) [17], a pattern attributed in part to rapid population ageing (median age of 45.7 years versus 41.2 years nationally) [16] and also to higher rates of behavioural risk factors such as physical inactivity and unhealthy diet. Approximately 1 in 10 New Brunswickers (10.54% (95% CI: 10.46–10.61)) had used healthcare services for a mood or anxiety disorder in 2015/16, a rate somewhat higher than the national average (10.05% (95% CI: 10.04–10.06)) [17]. Consistent with broadly established epidemiological trends, the provincial rate was significantly greater among females (13.84% (95% CI: 13.72–13.95)) than among males (7.15% (95% CI: 7.06–7.23)) [17], possibly reflecting sex-specific differences in healthcare seeking behaviours, detection, and treatment for mental health conditions.

### 2.2. Data Sources

Person-level provincial administrative health datasets were linked longitudinally with area-based socioenvironmental datasets for all residents. The pseudonymized administrative datasets included annual case ascertainments for AMI diagnoses, case ascertainments for use of physician or hospital services for diagnosed mood and anxiety disorders, vital statistics death records, and resident registrations and eligibility for New Brunswick Medicare, the publicly funded insurance program covering all essential physician and hospital services. Record linkages were performed deterministically using patients’ (pseudonymized) Medicare numbers. Because of the universality of healthcare coverage for all medically necessary services, the data comprised a complete enumeration of the eligible population (apart from full-time members of the Canadian Forces and federal inmates, who have federal healthcare coverage). The administrative health datasets were made available for research use through a legislated data sharing agreement with the Government of New Brunswick in the secure computing environment of the New Brunswick Institute for Research, Data and Training (NB-IRDT), located at the University of New Brunswick [18]. Ethics approval for this study was obtained from the Research Ethics Board of the University of New Brunswick (REB #2017-076), as part of a larger investigation into factors affecting chronic disease prevention and management using linked administrative datasets.

Based on annual residential postal code information, each individual was assigned several neighbourhood-level indicators of socioenvironmental characteristics (described in detail below) from datasets made available through the Canadian Urban Environmental Health Research Consortium (CANUE). These datasets include standardized indicators for every postal code in Canada on information such as land use and socioeconomic conditions [19]. Residents of New Brunswick are required to update the residential address associated with their Medicare card every time they move; a conversion file [20] was used to geocode six-digit postal codes to standard census geographies by fiscal year (with each fiscal year covering the period from April 1 of a given calendar year to March 31 of the following calendar year). In urban areas, the representative location of a six-digit postal code corresponds typically to one side of a street in a given block or the centre of an apartment building; in rural areas, there is much greater positional uncertainty (typically accurate within a few kilometres) [21].

### 2.3. Study Population

The study population included all adults aged 45 years and older alive after a heart attack and residing in New Brunswick; to ensure sufficient sample size, five years of data (2003/04 to 2007/08 fiscal years) were pooled together to establish a baseline cohort. These individuals were then followed over an average 11-year period of observation (that is, to the end of the 2015/16 fiscal year). Patients were censored at the time of death or departure from the province, as per the vital statistics death database and resident registry, respectively. Case ascertainments of AMI diagnoses were based on a validated algorithm for hospitalization data from the Canadian Chronic Disease Surveillance System (CCDSS), established among federal, provincial, and territorial partners to produce population-based estimates of chronic disease prevalence and incidence using administrative health data [1,22]. Diagnosed AMI cases were captured according to the coding standards of the Canadian adaptation to the International Classification of Diseases (ICD-10-CA codes I21–I22) [23]. Several studies have assessed the validity of hospitalization data for identifying myocardial infarction to be high (sensitivity and specificity ≥ 86% and positive predictive value ≥ 93%) [24].

### 2.4. Health Service Contacts for Mood and Anxiety Disorders

The outcome of interest was patients’ use of healthcare services, including visits to a physician (or nurse practitioner) or hospital admission, at least once in a given year for a mood or anxiety disorder. While there are many kinds of mental illnesses, these are the most common in Canada and worldwide, and include depression, generalized anxiety disorder, social anxiety disorder, and other disorders usually accompanied by a change in mood, symptoms, and activities that interfere with an individual’s everyday life [25]. Ascertainments of mood and anxiety disorders were identified from provincial administrative health databases drawing on the CCDSS infrastructure and case definitions (ICD-10-CA codes F30–F42, F44–F48, and F68) [23]. Excluded from the analysis were conditions with a demonstrably different etiology such as dementias, sleep disorders, and substance use disorders. Administrative data may not capture all cases of mental illness, or may capture cases that do not meet all standard diagnostic criteria for mood and anxiety disorders [25]. A triangulation analysis of prevalence estimates for mood and anxiety disorders using CCDSS data versus national household survey findings indicated there has been increasing consistency of information over time, reinforcing data validity [26].

### 2.5. Neighbourhood Socioenvironmental Characteristics

The health implications for seven different indicators of patients’ neighbourhood environments were considered. These included four indicators of socioeconomic characteristics (i.e., material deprivation, residential instability, ethnic concentration, population dependency) and three indicators of physical characteristics (i.e., active living friendliness, community size, and local climate zone), each of which is described below. First, we assigned to patients four dimensions of marginalization from the Canadian Marginalization Index (CAN-Marg), which was developed by Matheson et al. [27] to examine aspects of economic inequality associated with adverse health and healthcare outcomes. The CAN-Marg dataset includes four area-based indicators: material deprivation (e.g., proportion of low-income families, homes needing major repair, unemployment rate); residential instability (e.g., level of crowding, residential ownership, residential mobility); population dependency (e.g., labour force participation rate, proportion of seniors); and ethnic concentration (proportions of recent immigrants and visible minorities). These variables were developed at the scale of census dissemination areas, which are the smallest units of census geography, covering populations of approximately 400 to 700 people. The CAN-Marg measures have been found to be temporally and spatially stable across Canada, and to be associated with health status and health behaviours [28,29]. The lowest two quintiles of CAN-Marg values were defined here as socioeconomically marginalized neighbourhoods.

Next, patients were assigned values from the Canadian Active Living Environments (Can-ALE) dataset. The Can-ALE index summarizes features of communities that may support walkability and active living, such as densities of homes, green spaces (e.g., parks), footpaths, and transit stops [30]. Similar to the CAN-Marg data, this areal measure is based on 2006 census information by dissemination area. After assigning an index value to each individual’s place of residence for each fiscal year, the lowest quintile of Can-ALE values was defined as the least favourable active living environment.

Patients’ place of residence was then categorized by community size: urban versus rural. As defined for purposes of provincial healthcare resource planning, residences were considered “rural” if they were located more than 40 km outside of one of New Brunswick’s three main cities (Moncton, Fredericton, or Saint John). Urban residences were those located within the catchment areas of the three cities, each of which has a population under 150,000.

Lastly, given that there can be great variability in neighbourhood characteristics across these two broad categories of community size, and because the densities of dwellings and intersections (as described by the Can-ALE index) do not completely encapsulate the walkability or active living friendliness of different neighbourhoods, the physical characteristics of local environments were described further. For this, the recently developed local climate zone (LCZ) classification system was used, which incorporates a combination of characteristics related to land cover and land use for human activity [31,32]. The LCZ system classifies dissemination areas according to “built” and “natural” zones taking into account, among other details, building and vegetation types and heights, building and tree spacing, fraction of sky visible from ground level, proportion of surface with impervious cover, and areas of natural forests and wooded recreation, as derived from land cover/land use maps and satellite images [32]. Zones range from those characterized by a very dense mix of tall buildings that is mostly paved and has few trees (e.g., the compact high-rise design of downtown Toronto) to those characterized by a sparse arrangement of mostly single-story buildings in a more natural setting (e.g., the lightweight low-rise setting of a small, rural community). This system was designed to be applicable universally to urban environments worldwide and has the potential to classify human settlements in support of applications such as disaster mitigation, urban planning, and population assessment [33]. In the New Brunswick context, given the absence of the lowest LCZ values on the classification’s 20-point scale (i.e., the most compact high-rise areas), areas were grouped into those with lower LCZ values (13 or less) versus those with higher LCZ values (14 or higher). Similar to the other CANUE datasets, this index was developed drawing on census information for the year 2006, which represents approximately the midpoint of the study period.

### 2.6. Statistical Analysis

Cox proportional hazard models were used to assess the associations between health service contacts for mood and anxiety disorders and each of the seven area-level characteristics. To control for recent history of common mental disorders, patients having used mental health services in the five years preceding the AMI (that is, based on retrospective data from 1998/99 to 2006/07, depending on the time of cohort entry) were excluded from the analysis. For all models, individuals’ age was included as a time-varying control variable and sex as a time-invariant control variable over the period of observation. To see if there was a sex-specific difference in the socioenvironmental correlates of mental health risks among AMI patients, separate models for men and women were also analyzed. Hazard ratios (HRs) and bootstrapped 95% confidence intervals (CIs) were generated for each predictor using the Stata v15 statistical software package. Population counts were rounded to a base of five to reinforce the confidential nature of the administrative health data.

## 3. Results

**Figure 1:**
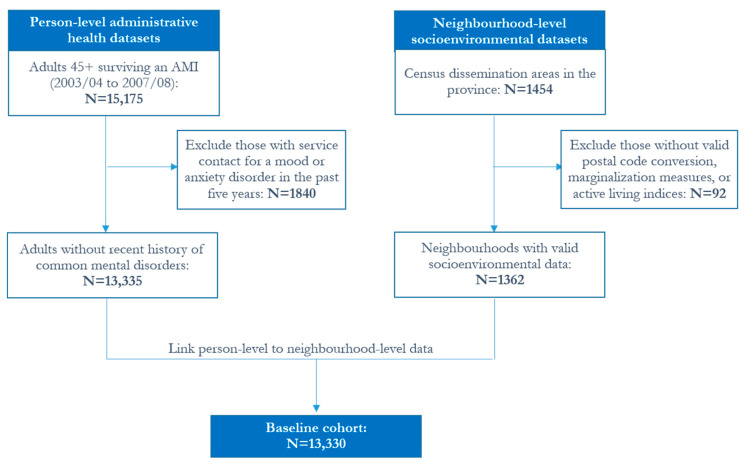
Flow chart for the creation of the cohort analysis file from linked administrative health and geo-environmental datasets. Note: Counts may not add up due to controlled rounding. AMI: acute myocardial infarction.

Among New Brunswickers aged 45 and over, 15,175 survived an AMI at any time over the baseline period (2003/04 to 2007/08 fiscal years) and had complete residential history information in the province over the average 11-year follow-up period of observation (that is, until the end of 2015/16); after excluding individuals with a recent history of prevalent mental disorders, the cohort for analysis included 13,330 adults residing in one of 1362 neighbourhoods (Figure 1).

**Figure 2:**
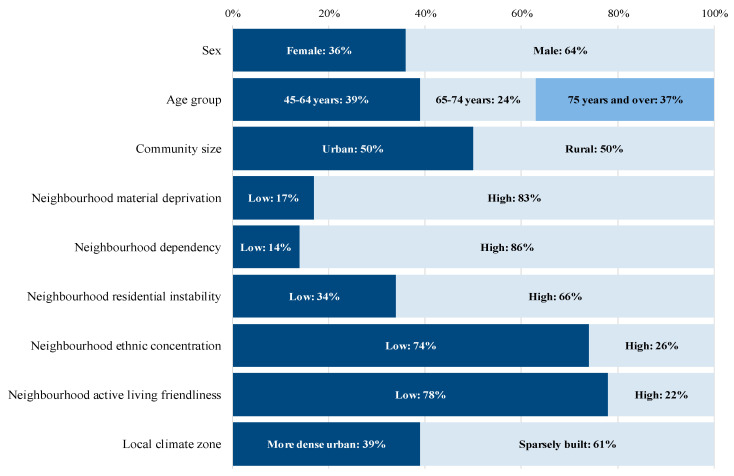
Percentage distribution of the adult population aged 45 and over with prior AMI by individual and neighbourhood-level characteristics, New Brunswick, Canada. Note: Individual and area-based characteristics are those at the time of patient entry to the cohort (N = 13,330 over the baseline period 2003/04 to 2007/08).

Of the baseline cohort of AMI survivors, 64.3% were men and 35.7% were women. At least two-thirds were living in neighbourhoods characterized by high material deprivation, low active living friendliness, high neighbourhood population dependency, high neighbourhood residential instability, and low neighbourhood ethnic concentration (Figure 2). Although half (50.5%) were living in urban communities, in the New Brunswick context only 38.9% were living in areas categorized as more compact high-rise urban designs.

Over the period of observation, 4330 (32.5%) of the AMI patient cohort used healthcare services at least once for a diagnosed mood or anxiety disorder (not shown). As expected, this proportion was higher among women (36.4%) compared to men (30.3%).

Table 1 presents the results from the hazard models for the associations between healthcare use for mood and anxiety disorders and each of the neighbourhood characteristics. Findings showed significantly increased risk of using health services for mood and anxiety disorders among AMI patients aged 45 and over living in neighbourhoods characterized by high ethnic concentration (HR: 1.11 (95% CI: 1.03–1.19), *p* < 0.05) after adjusting for age, sex, and size of home community. Other characteristics of local environments, including material deprivation and residential instability, were not found to be significantly associated with health service contacts for mental health disorders. As expected, the risk was lower among adults aged 75 years and over compared to their younger counterparts. The patient’s sex was significantly associated with the outcome of interest, with females having approximately 58% (HR: 1.58 (95% CI: 1.48–1.68), *p* < 0.05) higher risk of healthcare use for a mood or anxiety disorder compared to males, after controlling for age and the different measures of neighbourhood environments.

Among women alone (Table 1, model 2), and after adjusting for age and size of home community, neighbourhood-level material deprivation was significantly associated with increased risk of health service use for common mental illnesses (HR: 1.16 (95% CI: 1.01–1.33), *p* < 0.05). Among males alone (Table 1, model 3), only neighbourhood ethnic concentration was found to be independently associated with increased mental health service use (HR: 1.14 (95% CI: 1.03–1.25), *p* < 0.05).

## 4. Discussion

This study represented the first assessment of the role of neighbourhood environments on the burden to a publicly funded healthcare system for mental health comorbidities among patients recovering from AMI. Drawing on linked person-level administrative and geospatial datasets tracking individuals’ healthcare service contacts over an average 11-year period, and controlling for prior records of mental disorders, selected socioenvironmental characteristics of local communities were found to be significantly associated with greater risk of healthcare use for mood and anxiety disorders among post-AMI patients aged 45 and over in the province of New Brunswick, one of Canada’s most rural populations. However, the impact of specific neighbourhood characteristics varied by sex. Among men aged 45 and over, neighbourhood ethnic concentration was significantly associated with increased mental health service use (HR: 1.14 (95% CI: 1.03–1.25)), whereas among women it was neighbourhood material deprivation that played an independent role (HR: 1.16 (95% CI: 1.01–1.33)).

The findings of this study are generally consistent with research elsewhere that has reported links between different measures of neighbourhood marginalization with the risk of depression among adults [34], and with the risk of hospital admission for depression and other mood and anxiety disorders among adults with a neurodegenerative disease [15]. Living in areas of higher ethnic concentration or higher material deprivation has been associated with increased social vulnerability and associated health-risk behaviours contributing to poorer physical and mental health, including physical inactivity, binge drinking, and tobacco use [27]. The influential role of neighbourhood ethnic concentration was ascertained even in this study setting of relative ethnic homogeneity. While there is growing recognition of the importance of considering neighbourhood-level exposures in health research, studies specifically looking at community influences on health among older adults are limited and, where available, have been largely cross-sectional in design [35]. In particular, the LCZ classification system has been largely limited to analyses of urban heat islands, but its value for applications to city planning is increasingly recognized [32] and how it relates to human health is a new area of research.

The present results further reinforce the need for better integration of sex and gender in population health research. For example, Petkovic et al.’s analysis of recent systematic reviews documented that less than 30% of reviews reported on sex or gender in the results [36]. In this study, given that men numerically outweighed women in the target population (as expected), the statistical models including both sexes revealed only those substantive patterns in which neighbourhood characteristics were associated with poorer health outcomes that were predominantly observed among men (i.e., neighbourhood ethnic concentration). Using a split-sex analysis amplified cautions that unless explicit attention is paid to sex and gender in study designs, decisions based on the research evidence may inadvertently contribute to gender gaps by inadequately responding to different needs of disadvantaged women and men with chronic illness [37].

A certain underestimation of the prevalence of mental health service use in this study is likely. While increasing mental health literacy and declining stigma towards mental health disorders may be resulting in increased service use and improved recording of treatment over time [26], the administrative health datasets used here lacked information on mental health service use from exclusively community-based settings, private settings, or outside the province. Also lacking were data on individual risk and protective health-related behaviours and individual socioeconomic position. The present research relied on area-based socioenvironmental measures, categorizing all patients residing within a given census-defined boundary in the year with the same set of neighbourhood-level indicators. Given that these indicators (from the CAN-Marg, Can-ALE, and LCZ datasets) were available only for the census year 2006, it was assumed that they remained relatively stable over the study period. In a study from another Canadian province using different neighbourhood walkability data during 12 years of follow-up, Creatore et al. [13] found that most neighbourhoods remained in the same quintile and 99% remained within one quintile of their baseline assignment. In the context of uniquely smaller urban and rural settlements such as New Brunswick, indicators of active living environments may be little more than a marker for the urban cores.

Notwithstanding the above-noted limitations, strengths of this study included the population-based nature of the data, which captured all adults in the province with prior AMI, controlling for recent records of common mental disorders, and considering many different dimensions of built environments and neighbourhood social and economic deprivation. The availability of annually updated residential postal codes allowed for incorporating time-varying contextual covariates in the statistical analyses.

## 5. Conclusions

Despite universal healthcare coverage, selected local characteristics of neighbourhood environments were found to be significantly associated with increased health service contacts for mental health comorbidities among AMI patients aged 45 and over residing in a Canadian province of uniquely smaller urban and rural settlements. Among women, living in a materially deprived neighbourhood was significantly associated with increased mental health service use; among men, living in a neighbourhood characterized by ethnic concentration exercised an independent influence. These findings suggest that strategies aiming to improve detection and treatment of mental disorders need to consider the different needs of women and men across socioenvironmental settings. Further research is needed to understand the role of community situations to improve mental health outcomes following an acute cardiac event in the context of population ageing.

## Figures and Tables

**Table 1:** Adjusted hazard ratios (HRs) and 95% confidence intervals (CIs) for associations between individual and neighbourhood characteristics and risk of healthcare use for mood and anxiety disorders among AMI patients aged 45 and over; total and by sex.

Characteristic		(1) All Patients (n = 13,330)			(2) Females (n = 4755)			(3) Males (n = 8580)		
		HR	Lower CI	Upper CI	HR	Lower CI	Upper CI	HR	Lower CI	Upper CI
**Sex**										
	Female (ref: Male)	1.58 *	1.48	1.68	--	--	--	--	--	--
**Age group**										
	65–74 years (ref: 45–64 years)	0.95	0.88	1.03	0.91	0.80	1.03	0.97	0.88	1.07
	75+ years	0.94 *	0.87	1.00	0.81 *	0.72	0.91	1.05	0.96	1.16
**Community size**										
	Urban (ref: Rural)	0.91 *	0.85	0.97	0.95	0.85	1.06	0.88 *	0.80	0.96
**Neighbourhood material deprivation**										
	High (ref: Low)	1.03	0.95	1.12	1.16 *	1.01	1.33	0.95	0.85	1.06
**Neighbourhood population dependency**										
	High (ref: Low)	1.03	0.94	1.14	0.95	0.82	1.11	1.09	0.96	1.23
**Neighbourhood residential instability**										
	High (ref: Low)	1.04	0.97	1.11	1.03	0.92	1.15	1.04	0.95	1.13
**Neighbourhood ethnic concentration**										
	High (ref: Low)	1.11 *	1.03	1.19	1.07	0.95	1.20	1.14 *	1.03	1.25
**Neighbourhood active living friendliness**										
	High (ref: Low)	1.01	0.92	1.10	1.01	0.88	1.15	1.00	0.89	1.12
**Local climate zone**										
	Compact high-rise (ref: Lightweight low-rise)	1.01	0.94	1.08	1.07	0.96	1.19	0.97	0.89	1.05

* *p* < 0.05; ref = reference category. Counts may not add up due to controlled rounding.
